# Hydroxysteroid sulfotransferase 2B1 affects gastric epithelial function and carcinogenesis induced by a carcinogenic agent

**DOI:** 10.1186/s12944-019-1149-6

**Published:** 2019-11-22

**Authors:** Wenting Hong, Fenghua Guo, Mingjie Yang, Dongke Xu, Ziyan Zhuang, Baolin Niu, Qianming Bai, Xiaobo Li

**Affiliations:** 1 Department of Physiology and Pathophysiology, School of Basic Medical Sciences, Fudan University, Shanghai, 200032 China; 2 Department of General Surgery, Hua’shan Hospital, Fudan University Shanghai Medical College, Shanghai, China; 3 Department of Pathology, Fudan University Shanghai Cancer Centre, Shanghai, 200032 China; 4 Department of Oncology, Shanghai Medical College, Fudan University, Shanghai, China

**Keywords:** Gastric epithelial cell, Hydroxysteroid Sulfotransferase 2B1 (SULT2B1), Gastric carcinogenesis, Oxysterol, PI3K/AKT signaling

## Abstract

**Background:**

A healthy gastric mucosal epithelium exhibits tumor-suppressive properties. Gastric epithelial cell dysfunction contributes to gastric cancer development. Oxysterols provided from food or cholesterol oxidation in the gastric epithelium may be further sulfated by hydroxysteroid sulfotransferase 2B1 (SULT2B1), which is highly abundant in the gastric epithelium. However, the effects of SULT2B1 on gastric epithelial function and gastric carcinogenesis are unclear.

**Methods:**

A mouse gastric tumor model was established using carcinogenic agent 3-methylcholanthrene (3-MCA). A SULT2B1 deletion (SULT2B1^−/−^) human gastric epithelial line GES-1 was constructed by CRISPR/CAS9 genome editing system.

**Results:**

The gastric tumor incidence was higher in the SULT2B1^−/−^ mice than in the wild-type (WT) mice. In gastric epithelial cells, adenovirus-mediated SULT2B1b overexpression reduced the levels of oxysterols, such as 24(R/S),25-epoxycholesterol (24(R/S),25-EC) and 27-hydroxycholesterol (27HC). This condition also increased PI3K/AKT signaling to promote gastric epithelial cell proliferation, epithelization, and epithelial development. However, SULT2B1 deletion or SULT2B1 knockdown suppressed PI3K/AKT signaling, epithelial cell epithelization, and wound healing and induced gastric epithelial cell malignant transition upon 3-MCA induction.

**Conclusions:**

The abundant SULT2B1 expression in normal gastric epithelium might maintain epithelial function via the PI3K/AKT signaling pathway and suppress gastric carcinogenesis induced by a carcinogenic agent.

## Introduction

Gastric cancer is the fifth most common cancer worldwide and the second most common cancer in China [1–8]. Patients with gastric cancer generally have a poor prognosis, with a less than 30% 5-year survival rate in advanced stage [9]. The mechanism underlying gastric carcinogenesis remains to be elucidated.

Gastric mucosal epithelium controls important digestive, absorptive, and secretory functions and forms the first barrier against gastric juice, dietary irritants, and the microbiota. A healthy epithelium is composed of a polarized epithelial cell layer, which is maintained by the integrity of the apical-basal polarity, a highly organized actin cytoskeleton, and a junctional complex exhibiting tumor-suppressive properties [10]. The gastric epithelial cells of the gastric mucosa undergo dynamic changes, such as differentiation, growth, migration, and shedding. Impaired epithelial integrity may contribute to the carcinogenesis of gastric cancer [11]. Growth factors are involved in various aspects of epithelial homeostasis [6]. Epidermal growth factor (EGF) and insulin-like growth factor-1 (IGF-1) participate in the regulation of tight junction (TJ) proteins and wound healing via PI3K/AKT signaling pathway [3–8].

Oxysterols are derived from dietary or endogenous sources. The majority of endogenous oxysterol species are oxidized derivatives of cholesterol, such as 7α- and 7β-hydroxycholesterol (7αHC, 7βHC), 7-ketocholesterol (7KETO), 4β-hydroxycholesterol (4βHC), 24S-hydroxycholesterol (24(S)HC), 25- hydroxycholesterol (25HC), and 27-hydroxycholesterol (27HC). However, 24 (R/S),25-epoxycholesterol (24(R/S), 25-EC) is formed via a shunt of the mevalonate pathway in parallel to cholesterol synthesis [12–16]. Oxysterols are not only intermediates in the synthesis of bile acids and steroid hormones but also important regulators of genes involved in cholesterol and lipid metabolism. Oxysterols also may serve important functions in inducing inflammatory responses, cell survival, and differentiation [17].

In vitro studies, animal studies, and clinical investigations have suggested that oxysterols may play important roles in the pathophysiology of a wide spectrum of diseases, such as cancers and degenerative and age-related diseases. This role is rooted to their ability to trigger cell death, activate oxidation and inflammation, and modulate lipid homeostasis [18–21]. In addition, oxysterols may decrease the barrier functions of intestinal epithelia and develop an inappropriate inflammatory response to food compounds [22]. Oxysterols have complicated effects on different gastrointestinal cancers. Oxysterols exhibit pro-apoptotic/cytotoxic and pro-cancerous functions in colon tumor cells [23]. Oxysterols cooperate with ROS and lipid peroxides to cause metabolic disorders, DNA damage, and repair disorders; as a result, cell gene mutations that lead to the genesis of cholangiocarcinoma occurs [23–25]. The levels of 24(R/S),25-EC and 27HC in human gastric tumor tissues are significantly increased compared with those of adjacent normal gastric mucosal tissues. The levels of 24(R/S),25-EC, 25HC, 4βHC, 7αHC, and 7βHC in cancerous gastric juice are dramatically increased compared with gastric juice from healthy subjects [26].

Hydroxysteroid sulfotransferase 2B1 (SULT2B1) is a member of sulfotransferase that utilizes 3′-phospho-5’adenylyl sulfate as sulfonate donor to catalyze the sulfate conjugation of many hormones, drugs, neurotransmitters, and xenobiotic compounds. SULT2B1a and SULT2B1b, which result from alternative splicing of the SULT2B1 gene, are two isoforms of SULT2B1. SULT2B1b, which is the only SULT2B1 protein detected in human tissues or cell lines, is highly selective for the sulfation of 3β-hydroxysteroids, such as cholesterol, oxysterols, DHEA, D5-adiol, 5a-androstane-3β, 17β-diol (Anstane-diol), and pregnenolone [27–29]. SULT2B1 is expressed in high level in the stomach. However, the physiological and pathophysiological significance of SULT2B1 in the stomach and the possible role of SULT2B1 in gastric carcinogenesis are poorly understood.

Therefore, the ability of SULT2B1 to modulate oxysterol levels in the gastric epithelium may affect gastric epithelial cell function and gastric carcinogenesis. In the current study, the roles of SULT2B1 and oxysterols on gastric epithelial function were investigated, and the incidence of carcinogenic agent-induced gastric tumor was compared between wild-type (WT) and SULT2B1 deletion (SULT2B1^−/−^) mice.

## Materials and methods

### Clinical sample collection

Prior written informed consent was obtained from every patient, and the study was approved by the Ethics Review Board of Fudan University Affiliated Hua’shan Hospital (No. 2017–222). The normal gastric mucosal tissues at various sites (fundus, corpus, and antrum) were collected from 2 gastric cancer patients after total gastrectomy. The tumor tissue and adjacent normal gastric mucosal tissue specimens were collected during radical gastrectomies from 7 patients with gastric adenocarcinoma diagnosed and confirmed by pathological diagnosis and did not receive radiotherapy or chemotherapy. None of these patients was diagnosed with hyperlipidemia or diabetes.

### Mouse gastric carcinoma models

All animal experiments were performed with approval from the Fudan University School of Basic Medical Sciences Animal Ethics Committee. The SULT2B1^−/−^ mice were obtained from The Jackson Laboratory (https://www.jax.org/strain/018773). Mouse gastric carcinoma model was established via induction with the carcinogenic agent 3-methylcholanthrene (3-MCA) [30–32]. Briefly, the WT and SULT2B1^−/−^ mice (age 20–22 weeks) were anesthetized with isoflurane and the stomach was opened along the greater curvature of the anterior wall via laparotomy. A sterile cotton thread with a DMSO-impregnated knot containing approximately 1 mg of 3-MCA was inserted into the lesser curvature of antrum, from mucosa to serosa, through the greater curvature incision. In addition, another knot was tied to the serosal surface so that the two knots riveted the stomach wall. A figure-of-eight suture was carried out to embed the serosal surface knot. Finally, the stomach wall and abdominal incisions were sutured. Twelve weeks after the cotton thread was inserted, the mice were euthanized, and the mouse stomachs and nodules were fixed and processed for histopathological assessment of the cancerous lesions.

### Cell culture

The human normal gastric epithelial cell line GES-1 [33–35] was maintained in RPMI 1640 medium (Invitrogen, CA, USA) supplemented with 10% fetal bovine serum (Gibco, Invitrogen, USA) and 100 units/mL penicillin and streptomycin at 37 °C in a humidified incubator with 5% CO_2_. Human primary stomach epithelial cells (catalog no. H-6039) were purchased from Cell Biologics Company (IL, USA). The cells were maintained in human epithelial cell medium (catalog no. H6621, Cell Biologics Company).

### Infection of GES-1 with adenovirus encoding human SULT2B1b

GES-1 cells were infected with recombinant adenovirus encoding Ad-CMV-SULT2B1b at a multiplicity of infection (MOI) of 20 for 48 h as previously described [36]. The adenovirus encoding Ad-CMV-GFP was used as a control. The recombinant adenoviruses encoding Ad-SULT2B1b were prepared as previously described [37].

### Construction of the SULT2B1^−/−^ GES-1 cell line

To construct the SULT2B1 knockout GES-1 cell line, an online sgRNA design tool based on the clustered, regularly interspaced, short palindromic repeats (CRISPR)/CRISPR-associated protein 9 (CAS9) (CRISPR/CAS9) system (http://crispr.mit.edu/) was utilized. Two sgRNA oligos targeting exon 2 of the SULT2B1 gene were selected as follows: 5′-CGAGTACAGGCCGACGGGGAAGG-3′ (sgRNA-A) and 5′-CGGAGAACACCCAAGATGTGCGG-3′ (sgRNA-B). sgRNA-A and sgRNA-B were cloned into the pSpCas9n (BB)-2A-Puro vector (pX462 vector, Addgene plasmid # 48141) [38]. Then, the constructed plasmids containing sgRNA-A and sgRNA-B were co-transfected into GES-1 cells with Lipofectamine 3000 (Invitrogen, CA, USA). SULT2B1 gene knockout was confirmed by Western blotting and gene sequencing.

### siRNA interference and transfection

The cells were transfected with human SULT2B1-specific siRNA duplex oligo ribonucleotides, with a 5′-GATCGAGATCATCTGCTTA-3′ targeting sequence, or negative control duplexes (RiboBio, Guangzhou, China) using Lipofectamine 3000.

### cDNA library construction and RNA-seq analysis

Total RNA was extracted with TRIzol (Life Technologies, Thermo Fisher Scientific, USA). A TrueLib mRNA Library Prep Kit for Illumina (ExCell. Bio, Shanghai, China) was adopted for mRNA enrichment and cDNA library preparation. The library quality was assessed using an Agilent 2100 Bioanalyzer. Sequencing was performed on an Illumina HiSeq X Ten at Annoroad Gene Technology Co., Ltd. (Beijing, China).

The data of RNA-seq were analyzed as described previously [39]. The human genome sequence was obtained from the UCSC Genome Browser (GRCh38) (http://genome.ucsc.edu/). The annotated gene models were taken from Ensembl (http://www.ensembl.org/). The RNA-seq reads from the FASTQ files were mapped to the human reference genome (GRCh38), and the splice junctions were identified by TopHat. To estimate transcript abundance, the output files in binary alignment/map (BAM) format were analyzed by Cufflinks. The mRNA abundance was expressed in fragments per kilobase of exon per million reads mapped (FPKM). DAVID (http://david.abcc.ncifcrf.gov/home.jsp) functional annotation cluster analysis was employed to list differentially expressed genes (over 1.5-fold).

### Real-time quantitative polymerase chain reaction (qRT-PCR) analysis

Reverse transcription (RT) was performed using a PrimeScript RT reagent kit (Takara, Shiga, Japan) according to the manufacturer’s instructions. The relative expression levels of the target genes were determined by SYBR Premix Ex Taq II (Takara, Shiga, Japan). The tested genes were quantified using the comparative cycle threshold (CT) method, and the results were normalized to the 36B4 level. The primer sets used for gene expression analysis are shown in Table S3.

### Western blotting analysis

Samples were lysed in RIPA buffer (Beyotime, Shanghai, China) containing protease inhibitor cocktail, phosphatase inhibitors and phenylmethylsulfonyl fluoride (PMSF). Equal amounts of protein (30–50 μg) were loaded and separated on a 10% SDS-polyacrylamide gel, electrotransferred onto PVDF membranes (Millipore, Billerica, MA, USA), and blocked in 5% bovine serum albumin in Tris-buffered saline containing Tween 20 for 1 h at room temperature. The membranes were probed with primary antibodies against p-AKT (S473), AKT, CCND1, CCNA2, CLND1, CDH1 (Cell Signaling Technology, MA, USA), HSP90 (Proteintech, IL, USA) and SULT2B1 (R&D systems, MN, USA) overnight at 4 °C. After three 10 min washes with Tris-buffered saline with Tween-20 (TBST), the membrane was incubated at room temperature for 1 h with an appropriate secondary antibody conjugated to horseradish peroxidase. HSP90 was used as the loading control. The protein signals were visualized with a Pierce ECL Western Blotting kit (Life Technologies, Thermo Fisher Scientific, USA).

### Proliferation assay

A Cell-Light™ EdU Apollo 567 in vitro kit (RiboBio, Guangzhou, China) was used to determine the proliferative capacity of the cells. The cells (5 × 10^3^) were seeded onto the 96-well microplates and treated as indicated. 5-Ethynyl-2′-deoxyuridine (EdU) was added to the cell culture, and the cells were incubated for 2 h at 37 °C with 5% CO_2_. Afterwards, the cells were fixed with 4% paraformaldehyde and stained with Hoechst 33342 dye according to the manufacturer’s protocol. Then, the cells were observed under a fluorescence microscope. Cell fluorescence was measured with ImageJ software (National Institutes of Health, MD, USA).

### Immunofluorescence

Cells grown on glass coverslips were fixed with 95% ethanol for 20 min at 4 °C and washed 3 × 5 min with PBS. Cells were then blocked in 5% donkey serum/PBS for 30 min and then incubated with rabbit anti-Claudin-1 (Proteintech, IL, USA) in blocking buffer overnight at 4 °C. Cells were then washed 3 × 5 with PBS and then incubated with Alexa Fluor® 594 donkey anti-rabbit IgG. Nuclear DNA was stained by DAPI for 10 min. Cells on the glass coverslips were mounted with antifade mounting medium (Beyotime, Shanghai, China). The fluorescence of the cells was examined by confocal microscopy.

### Histopathology and hematoxylin and eosin (H&E) and immunohistochemical staining

After fixation in 4% paraformaldehyde and paraffin embedding, the gastric samples were sliced into 5 mm-thick sections and stained with H&E. Tissue sections were incubated with anti-SULT2B1 (R&D systems, MN, USA), anti-CKpan, anti-CK8, and anti-VIM antibodies (Cell Signaling Technology, MA, USA) and subsequently with a horseradish peroxidase-conjugated secondary antibody. The signals were detected using a diaminobenzidine substrate kit (Vector Laboratories, Burlingame, CA, USA), and the samples were counterstained with hematoxylin. Representative images were captured with a microscope (Leica DFC310 FX).

### Wound healing assay

GES-1 or SULT2B1^−/−^ cells were seeded onto the 6-well tissue culture plates. After transfection with Ad-GFP or Ad-SULT2B1b for 6 h, the monolayers were gently scratched with a pipette tip across the center of the well. After the scratch was made, the detached cells were removed by washing. Then the harvested cells were cultured in fresh medium for an additional 48 h. The photos of the same configurations were taken at the indicated times. The gap distance was quantitatively evaluated using ImageJ.

### Quantification of oxysterols by liquid chromatography-mass spectrometry (LC-MS)

LC-MS analysis was performed on oxysterols from the same number of pelleted treated cells (1 × 10^7^ cells) using an HPLC-quadrupole time-of-flight hybrid mass system according to the published methods [26, 39]. The typical total ion chromatograms (TIC) of samples are shown in Fig. S1, the multiple reaction monitoring (MRM) condition, quantitation ranges and linearity data are shown in Table S1, the limit of detection (LOD) and limit of quantification (LOQ) data are shown in Table S2.

### Statistical analysis

Experiments were repeated at least three times. Measurable data were expressed as means ± SD. Two-way analysis of variance (ANOVA) was employed to determine statistical differences among time-series experiments while the student’s t-test was performed for two-sample comparisons. The Log-Rank test was used to determine the difference in survival probabilities between the experiment group and the control group. GraphPad Prism 6.0 Software (San Diego, CA, USA) was used for calculations and graphs. *P* < 0.05 was considered statistically significant.

## Results

### SULT2B1-deficient mice were susceptible to gastric tumors upon induction with the carcinogenic agent 3-MCA

**Fig. 1:**
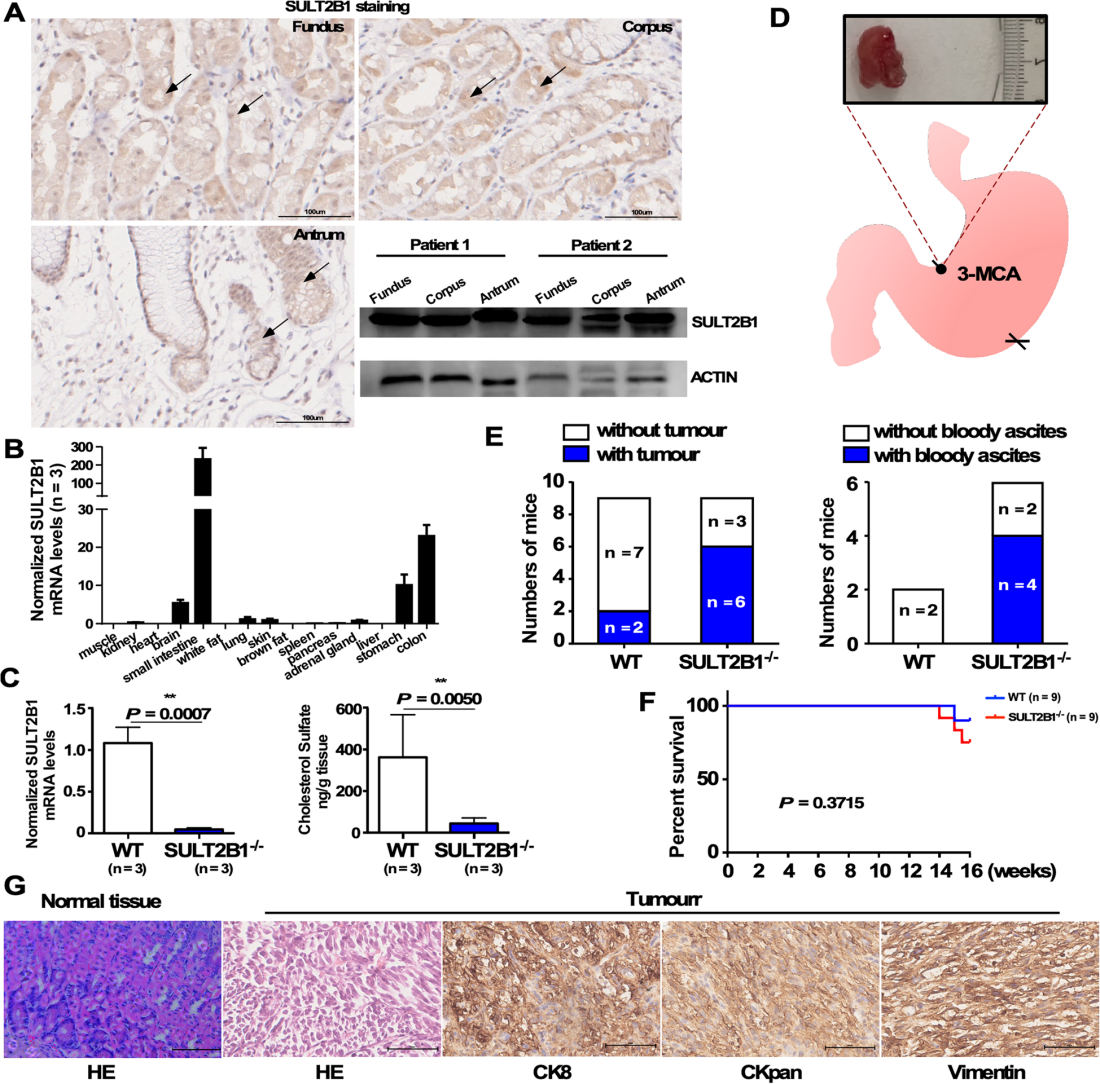
SULT2B1-deficient mice were susceptible to gastric tumors upon induction with the carcinogenic agent 3-MCA. **a** SULT2B1 expression in human normal gastric mucosal tissue was studied by immunohistochemical staining and Western blotting analysis. **b** The SULT2B1 mRNA level in different murine organs was detected by qRT-PCR. **c** The SULT2B1 mRNA expression level in the gastric tissues of WT and SULT2B1^−/−^ mice was measured by qRT-PCR. The concentration of cholesterol sulfate in the gastric tissues of WT and SULT2B1^−/−^ mice was detected by LC-MS. **d** The gastric carcinoma model was established by 3-MCA induction in WT and SULT2B1^−/−^ mice. **e** Three months after induction, the number of tumor-bearing mice with or without bloody ascites in the WT and SULT2B1^−/−^ mice was plotted. **f** The survival probabilities of WT and SULT2B1^−/−^ mice upon 3-MCA treatment. **g** The results from the H&E staining and immunohistochemical staining for CK8, CKpan, and VIM expression in the nodules are presented. * *P* < 0.05, ** *P* < 0.01

In the fundus, corpus and antrum of the human stomach, SULT2B1 was highly expressed in the gastric epithelium as evidenced by immunohistochemical staining and Western blotting analysis. SULT2B1 was mainly expressed in the cytoplasm of gastric epithelial cells (Fig. 1a). As shown in Fig. 1b, SULT2B1 mRNA was highly expressed in mouse small intestine, colon, brain and stomach. Thus far, the significance of the abundant SULT2B1 expression in the gastrointestinal tract has not been clarified. Although both SULT2B1a and SULT2B1b could be detected in human gastric epithelial cells (data not shown), only the SULT2B1b protein, not the SULT2B1a protein, could be detected in human tissues [28]. SULT2B1^−/−^ mice were used to investigate the role of SULT2B1 in gastric carcinogenesis. The mRNA level of SULT2B1 and the concentration of cholesterol sulfate (the product of SULT2B1) in the stomach were much lower in the SULT2B1^−/−^ mice than those in the WT mice (Fig. 1c). A gastric carcinoma model in WT and SULT2B1^−/−^ mice was established by induction with the carcinogenic agent 3-methylcholanthrene, as described in the Materials and Methods. As shown in Fig. 1d and e, at 3 months after induction, nodules (diameter ranges from 5 mm to 13 mm) were found in the lesser curvature of the antrum in 67% of the SULT2B1^−/−^ mice. All nodules invaded beyond serosa or adjacent structures. Bloody ascites were found in 67% of the tumor-bearing SULT2B1^−/−^ mice. However, nodules (with diameters of 3 to 5 mm) were found in only 22% of the WT mice, and bloody ascites were not observed. By the end of the observation, there was no significant difference in the survival probabilities between SULT2B1^−/−^ mice and WT mice (Fig. 1f). H&E staining and positive CK8, CKpan, and VIM staining suggested that the tumors were sarcomatoid carcinomas (Fig. 1g).

### RNA-seq analysis indicated that SULT2B1 was involved in epithelial development

**Fig. 2:**
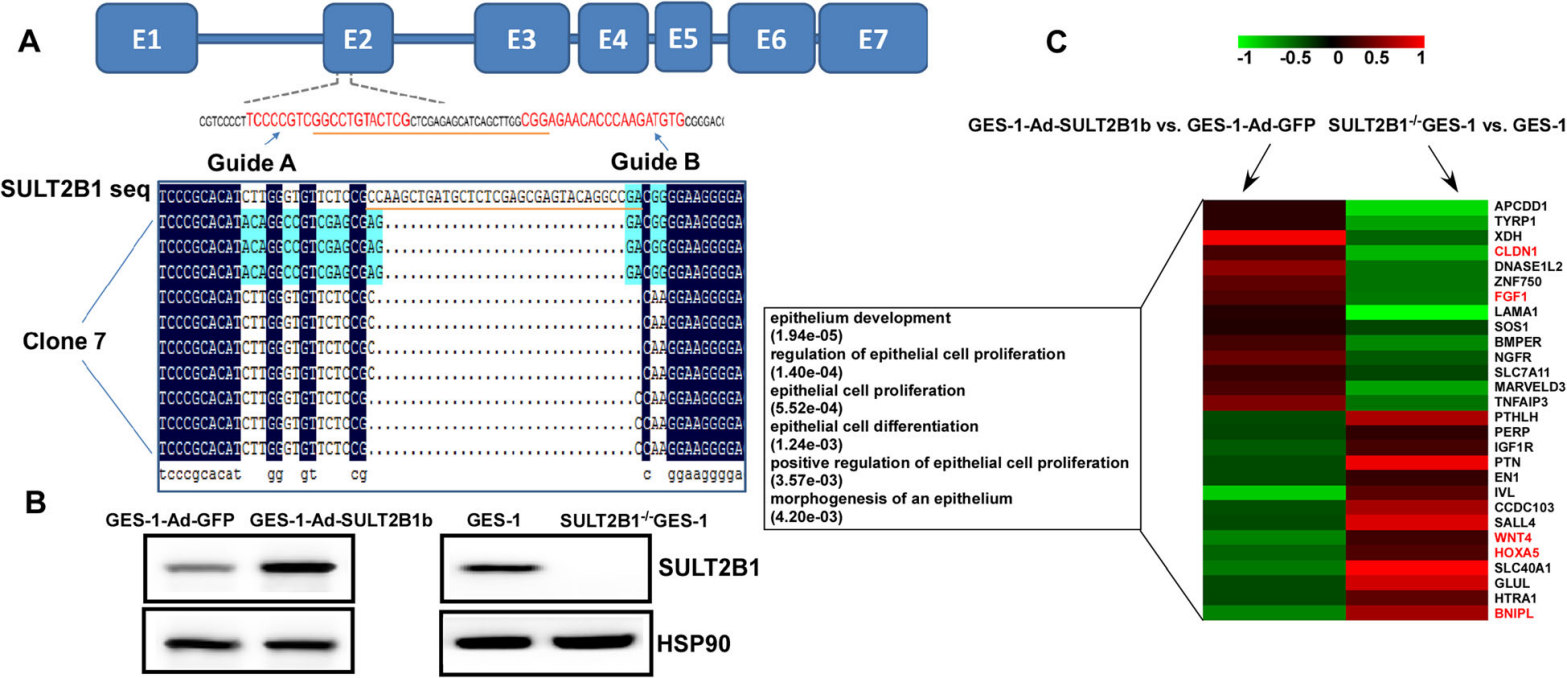
RNA-seq analysis indicated that SULT2B1 is involved in epithelial development. **a** Schematic diagram of the SULT2B1 gene (blue bars, exons) and the CRISPR/CAS9 gRNA sequence (guide **a** and **b**, red). TA cloning results of clone 7 showed that both alleles of the human SULT2B1 gene were modified on exon 2 by the CRISPR/CAS9 system. **b** The SULT2B1^−/−^ GES-1 cell line was established by the CRISPR/CAS9 technique. SULT2B1 protein expression was measured by Western blotting analysis in GES-1 cells, SULT2B1^−/−^ GES-1 cells, and GES-1 cells transfected for 48 h with Ad-GFP or Ad-SULT2B1b. **c** Among the genes changed by both SULT2B1b overexpression and SULT2B1 deletion, some genes in epithelial development were enriched. The fold changes in these gene levels are shown by the heatmap

To investigate the function of SULT2B1 in gastric epithelial cells, a SULT2B1 knockout human gastric epithelial line GES-1 was established by using CRISPR/CAS9 genome editing [40]. As shown in Fig. 2a, the double gRNAs specifically targeting exon 2 of the human SULT2B1 gene and subcloned it into the PX462 CRISPR/CAS9 vectors. GES-1 cells were transfected with the subcloned CRISPR/CAS9 plasmids. Single colonies with successful genome editing were selected by puromycin and Sanger’s sequencing at the cutting site on exon 2 of the SULT2B1 gene. To further determine whether both SULT2B1 gene alleles were modified in these clones, TA cloning of the PCR products flanking the CRISPR/CAS9 cutting site was performed. Clone 7 was selected because all three alleles of clone 7 were edited at exon 2 of the SULT2B1 gene. The SULT2B1 protein was undetectable by Western blotting analysis in this clone (Fig. 2b). Therefore, clone 7 was used for further study as the SULT2B1^−/−^ GES-1 cell line. Adenovirus-mediated SULT2B1b overexpression was also performed in GES-1 cells, which was confirmed by Western blotting (Fig. 2b).

Global transcriptome profiles between GES-1-Ad-GFP and GES-1-Ad-SULT2B1b groups and between GES-1 and SULT2B1^- / -^ GES-1 groups were compared by RNA sequencing and bioinformatics analysis. GO analysis was performed using DAVID’s default parameters. A total of 481 genes were upregulated and 706 genes were downregulated (over 1.5-fold) in the GES-1-Ad-SULT2B1b group compared with the GES-1-Ad-GFP group. Compared with the GES-1 group, the SULT2B1^−/−^ GES-1 group presented 1068 upregulated genes and 971 downregulated genes (over 1.5-fold change). There are 113 overlapping genes that were upregulated by SULT2B1b overexpression and simultaneously downregulated by SULT2B1 deletion. On KEGG pathway enrichment analysis, the 113 genes were enriched in the “PI3K-AKT signaling pathway” category. Among the genes altered by both SULT2B1b overexpression and SULT2B1 deletion, the genes involved in epithelium development are shown in Fig. 2c heatmap. Key genes, such as CLDN1 and FGF1, that facilitate epithelial development were increased in the Ad-SULT2B1b-treated epithelial cells but decreased in the SULT2B1^−/−^ GES-1 cells. Among the genes that decreased in the Ad-SULT2B1b-treated epithelial cells but increased in the SULT2B1^−/−^ GES-1 cells, BNIPL is involved in epithelial cell proliferation inhibition, and WNT4 and HOXA5 are involved in intestinal epithelial cell development.

### SULT2B1b overexpression promoted gastric epithelial cell proliferation via the PI3K/AKT signaling pathway

**Fig. 3:**
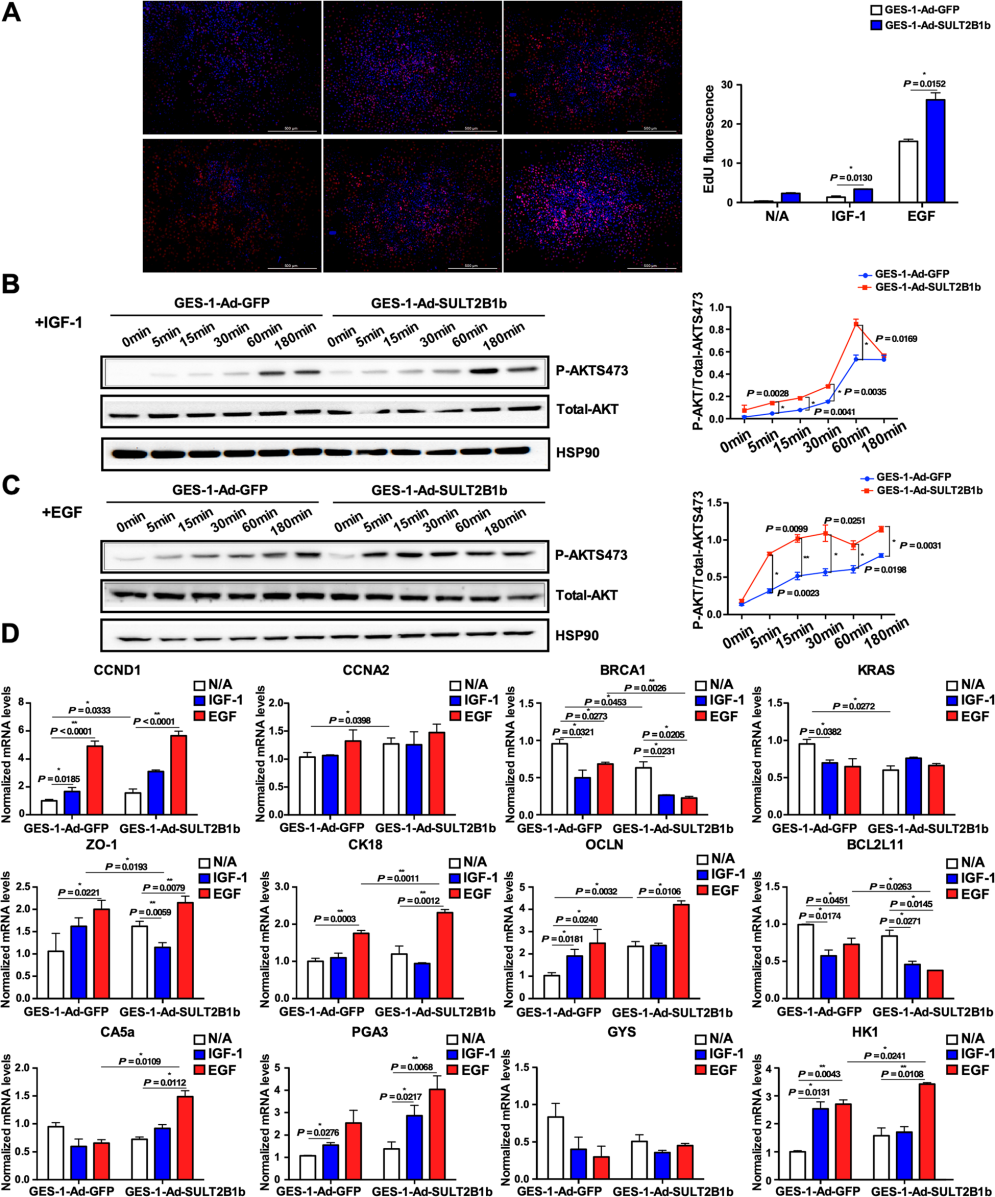
SULT2B1b overexpression promoted gastric epithelial cell proliferation via the PI3K/AKT signaling pathway. **a** GES-1 cells were treated with Ad-GFP or Ad-SULT2B1b for 48 h and then with IGF-1 (100 ng/mL) or EGF (100 ng/mL) for an additional 24 h. Cell proliferation was detected by the EdU incorporation assay. EdU fluorescence was normalized to that of Hoechst 33342. **b**, **c** After transfection with Ad-GFP or Ad-SULT2B1b for 48 h, GES-1 cells were stimulated with IGF-1 (100 ng/mL, **b**) or EGF (100 ng/mL, **c**) for 5, 15, 30, 60, or 180 min. AKT phosphorylation levels were detected by Western blotting. The p-AKT/t-AKT ratios are plotted. **d** After transfection with Ad-GFP or Ad-SULT2B1b for 48 h, GES-1 cells were stimulated with IGF-1 (100 ng/mL) or EGF (100 ng/mL) for 8 h. The mRNA levels of the genes involved in gastric epithelial function were measured by qRT-PCR. * *P* < 0.05, ** *P* < 0.01

IGF-1 and EGF promote gastric epithelial cell proliferation and development via the PI3K/AKT signaling pathway. The above RNA-seq data suggested that the PI3K/AKT signaling pathway is influenced by SULT2B1b expression. Therefore, to study whether SULT2B1b influences cell proliferative capabilities and PI3K/AKT signaling, SULT2B1b was overexpressed in GES-1 cells in the absence or presence of IGF-1/EGF. The GES-1 cells were treated with Ad-GFP or Ad-SULT2B1b for 48 h and then with IGF-1 (100 ng/mL) or EGF (100 ng/mL) for an additional 24 h. Cell proliferation was detected by EdU incorporation assays. As shown in Fig. 3a, proliferating cells have red nuclei whereas the nonproliferating cells appear to have blue nuclei. IGF-1 and EGF stimulation substantially increased GES-1 cell proliferation, which was further augmented by SULT2B1b overexpression. The PI3K/AKT signaling was further evaluated after IGF-1 or EGF treatment for 5, 15, 30, 60 or 180 min. As shown in Fig. 3b and c, the phosphorylation levels of AKT were significantly increased by SULT2B1b overexpression. Furthermore, the mRNA levels of downstream PI3K/AKT signaling genes were measured. GES-1 cells were transfected with Ad-GFP or Ad-SULT2B1b; after 48 h, the cells were treated with IGF-1 (100 ng/mL) or EGF (100 ng/mL) for an additional 8 h. As shown in Fig. 3d, IGF-1 and especially EGF increased the mRNA levels of proliferation-promoting genes CCND1 and CCNA2, epithelial markers ZO-1, CK18, and OCLN, and functional genes CA5a, PGA3 and HK1, whereas the mRNA levels of proliferation-suppressing genes BRCA1, KRAS, and BCL2L11 and functional gene GYS were reduced by IGF-1/EGF. The above effects were augmented by SULT2B1b overexpression. The mRNA levels of CCND1, CCNA2, ZO-1, CK18, OCLN, CA5a, PGA3 and HK1 increased, while the BRCA1, KRAS, BNIPL, BCL2L11 and GYS mRNA levels decreased in the GES-1-Ad-SULT2B1b cells compared with the GES-1-Ad-GFP cells in the absence or presence of IGF-1/EGF.

### SULT2B1 deletion suppressed PI3K/AKT signaling and epithelial function in GES-1 cells and contributed to transformation upon 3-MCA induction

**Fig. 4:**
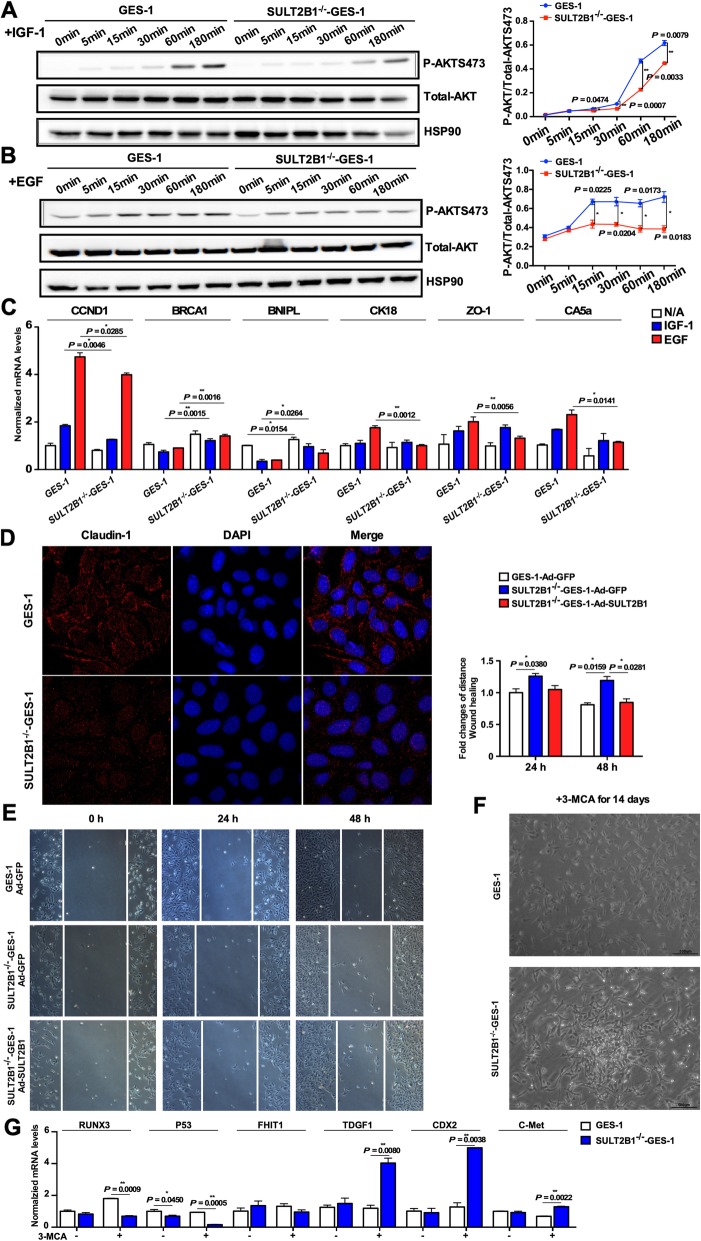
SULT2B1 deletion suppressed PI3K/AKT signaling in GES-1 cells and contributed to transformation upon 3-MCA induction. **a**, **b** The GES-1 and SULT2B1^−/−^ GES-1 cells were stimulated with IGF-1 (100 ng/mL, **a**) or EGF (100 ng/mL, **b**) for 5, 15, 30, 60, or 180 min. The phosphorylation levels of AKT were detected by Western blotting. The p-AKT/t-AKT ratios are plotted. **c** GES-1 and SULT2B1^−/−^ GES-1 cells were stimulated with IGF-1 (100 ng/mL) or EGF (100 ng/mL) for 8 h. The mRNA levels of the genes involved in gastric epithelial function were measured by qRT-PCR. **d** Immunofluorescent staining of claudin-1 [36] in GES-1 and SULT2B1^−/−^ GES-1 cells. The nuclei were revealed by DAPI staining (blue). **e** Wound healing assay of GES-1, SULT2B1^−/−^ GES-1 and SULT2B1^−/−^ GES-1 cells transfected with Ad-SULT2B1b. **f** GES-1 and SULT2B1^−/−^ GES-1 cells were treated with 3-MCA (2 μg/mL) for 14 days. 3-MCA was added into the medium every 2–3 days. The cell morphology is presented. **g** The mRNA levels of pro- and anticancerous genes were detected by qRT-PCR in GES-1 and SULT2B1^−/−^ GES-1 cells in the absence or presence of 3-MCA treatment for 14 days. * *P* < 0.05, ** *P* < 0.01

To further investigate the effect of SULT2B1 on gastric epithelial cell function, PI3K/AKT signaling and the expression of downstream genes following IGF-1/EGF treatment in GES-1 and SULT2B1^−/−^ GES-1 cells were evaluated. As shown in Fig. 4a and b, phosphorylated AKT levels decreased in the SULT2B1^−/−^ GES-1 cells compared with the GES-1 cells following either IGF-1 (100 ng/mL) or EGF (100 ng/mL) stimulation. The CCND1, CK18, ZO-1 and CA5a mRNA levels decreased, while the mRNA levels of BRCA1 and BNIPL increased in the SULT2B1^−/−^ GES-1 cells compared with the GES-1 cells in the absence or presence of IGF-1/EGF for 8 h (Fig. 4c). Immunofluorescence staining revealed that the expression of the tight junction marker Claudin-1 in SULT2B1^−/−^ GES-1 cells was lower than in GES-1 cells (Fig. 4d). As shown in Fig. 4e, the wound healing ability in SULT2B1^−/−^ GES-1 cells were lower than that in GES-1 cells, which could be rescued by SULT2B1b overexpression.

Since the SULT2B1^−/−^ mice were susceptible to gastric cancer upon 3-MCA induction, the tumorigenesis transformation tendencies of the GES-1 and SULT2B1^−/−^ GES-1 cells upon 3-MCA (2 μg/m) induction were compared. As shown in Fig. 4f, after 14 days of 3-MCA treatment, compact cell aggregate cultures appeared in the SULT2B1^−/−^ GES-1 cell dish but not in the GES-1 cell dish. After 14 days of 3-MCA induction, the mRNA levels of procancerous genes such as TDGF1, CDX2 and C-Met significantly increased, while the levels of the anticancerous genes RUNX3, P53 and FHIT1 decreased in the SULT2B1^−/−^ GES-1 cells. This change was greater in the SULT2B1^−/−^ cells than in the GES-1 cells (Fig. 4g).

### 24(R/S),25-EC and 27HC regulate gastric epithelial function

**Fig. 5:**
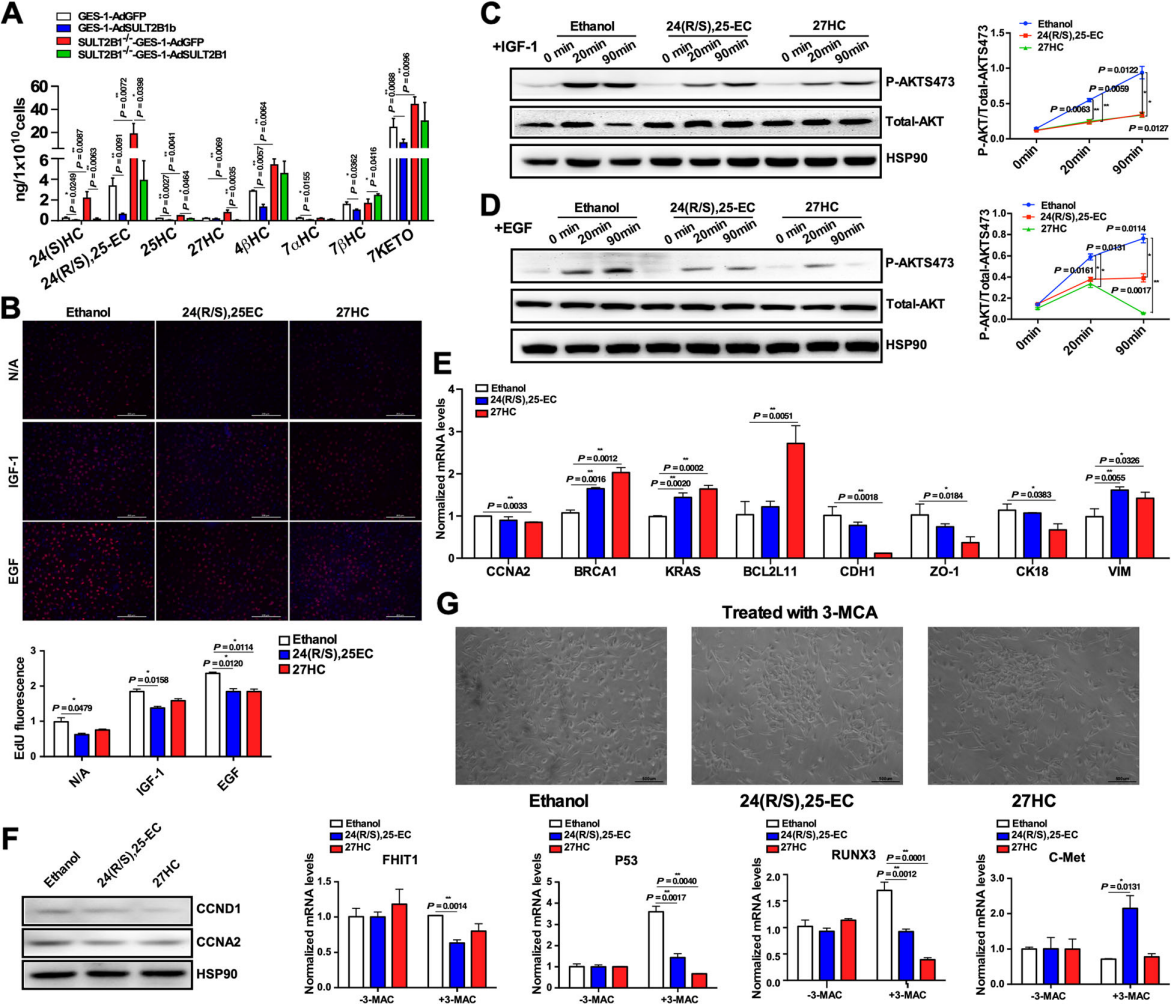
24(R/S),25-EC and 27HC regulate gastric epithelial function. **a** The levels of 8 oxysterols (25HC, 27HC, 24(S) HC, 24(R/S),25-EC, 7αHC, 7βHC, 4βHC and 7KETO) were simultaneously detected by LC-MS in GES-1 and SULT2B1^−/−^ GES-1 cells with or without adenovirus-mediated SULT2B1b overexpression. **b** The GES-1 cells were pretreated with 1 μmol/L of 24(R/S),25-EC and 27 HC for 12 h and then with IGF-1 (100 ng/mL) or EGF (100 ng/mL) for an additional 24 h. Cell proliferation was detected by the EdU incorporation assay. EdU fluorescence was normalized to that of Hoechst 33342. **c**, **d** GES-1 cells were pretreated with 1 μmol/L of 24(R/S),25-EC and 27HC for 12 h and then with IGF-1 (100 ng/mL, **c**) or EGF (100 ng/mL, **d**) for 20 or 90 min. The phosphorylation levels of AKT were detected by Western blotting. The p-AKT/t-AKT ratios are plotted. **e** GES-1 cells were treated with 1 μmol/L 24(R/S), 25-EC and 27 HC for 12 h. The mRNA levels of the genes involved in gastric epithelial function were measured by qRT-PCR. **f** CCND1 and CCNA2 protein expression levels were detected by Western blotting. **g** GES-1 cells were treated with oxysterol (1 μmol/L) and 3-MCA (2 μg/mL) for 10 days. Oxysterol and 3-MCA were added into the medium every 2–3 days. The cell morphology is presented. The mRNA levels of pro- and anticancerous genes in the absence or presence of oxysterol/3-MCA treatment for 10 days were detected by qRT-PCR. * *P* < 0.05, ** *P* < 0.01

SULT2B1 might be responsible for sulfating oxysterols. To determine the mechanisms by which SULT2B1 regulates gastric epithelial function, the levels of 8 oxysterols (25HC, 27HC, 24(S) HC, 24(R/S),25-EC, 7αHC, 7βHC, 4βHC, and 7KETO) in GES-1 and SULT2B1^−/−^ GES-1 cells with or without adenovirus-mediated SULT2B1b overexpression were detected by LC-MS simultaneously. As shown in Fig. 5a, SULT2B1b overexpression significantly decreased the oxysterol concentration in GES-1 cells, while SULT2B1 deletion increased these levels. By using the EdU incorporation assay, the effect of the two most significant oxysterols (24(R/S),25-EC and 27HC) on GES-1 cell proliferation was examined and turns out that these two oxysterols inhibited GES-1 proliferation in the absence and presence of IGF-1/EGF (Fig. 5b). The phosphorylated AKT levels were suppressed by 24(R/S),25-EC or 27HC after stimulation with either 100 ng/mL IGF-1 (Fig. 5c) or 100 ng/mL EGF (Fig. 5d). The CDH1, CCNA2, CK18 and ZO-1 mRNA levels decreased, while the BRCA1, BCL2L11, VIM and KRAS levels increased with oxysterol treatment (Fig. 5e). The CCND1 and CCNA2 protein expression levels also decreased with oxysterol treatment (Fig. 5f). Using 3-MCA (2 μg/mL) as a carcinogenic agent, the effects of these oxysterols on the tumorigenesis transformation tendency of GES-1 cells was further observed. As shown in Fig. 5g, after 10 days of 3-MCA treatment, more compact cell aggregate cultures appeared in the dish with GES-1 cells treated with oxysterols than in the dish with the vehicle (ethanol) control-treated cells. At 10 days after 3-MCA induction, the mRNA levels of the procancerous gene C-met were increased by the 24(R/S),25-EC treatment, while the levels of the anticancerous genes RUNX3, FHIT1 and P53 were decreased by the oxysterol treatments.

### SULT2B1 knockdown and oxysterols suppressed PI3K/AKT signaling in human primary stomach epithelial cells

**Fig. 6:**
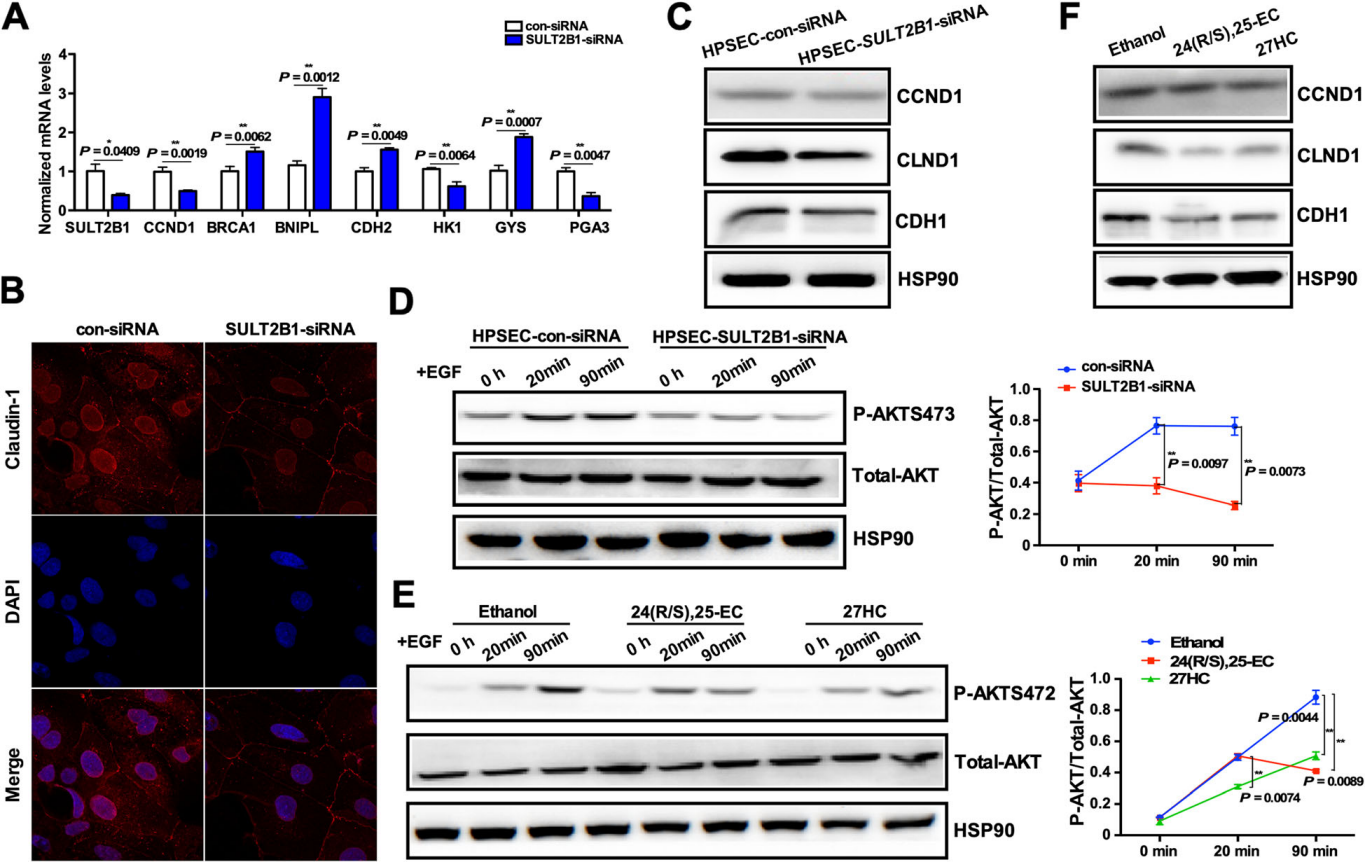
SULT2B1 knockdown and oxysterols suppressed PI3K/AKT signaling in human primary stomach epithelial cells. Human primary stomach epithelial cells (HPSECs) were transfected with control-siRNA or SULT2B1-siRNA for 48 h. **a** The mRNA levels of genes involved in gastric epithelial function were measured by qRT-PCR. **b** Immunofluorescent staining of claudin-1 [36] in HPSECs transfected with control-siRNA or SULT2B1-siRNA for 48 h. The nuclei were revealed by DAPI staining (blue). **c** The CCND1, CLND1 and CDH1 protein expression levels were detected by Western blotting. **d** After being transfected with control-siRNA or SULT2B1-siRNA for 48 h, the HPSECs were stimulated by EGF (100 ng/mL) for 20 or 90 min. The phosphorylation levels of AKT were detected by Western blotting. The p-AKT/t-AKT ratios are plotted. **e** The HPSECs were pretreated with 1 μmol/L of 24(R/S),25-EC or 27HC for 12 h and then treated with EGF (100 ng/mL for 20 or 90 min. The phosphorylation levels of AKT were detected by Western blotting. The p-AKT/t-AKT ratios are plotted. **f** HPSECs were treated with 1 μmol/L 24(R/S),25-EC and 27HC for 12 h. The CCND1, CLND1 and CDH1 protein expression levels were detected by Western blotting. * *P* < 0.05, ** *P* < 0.01

To further confirm the important role of SULT2B1 in gastric epithelial cells, SULT2B1 expression was silenced in human primary stomach epithelial cells (HPSECs). As shown in Fig. 6a, the CCND1, HK1 and PGA3 mRNA levels decreased, while the mRNA levels of BRCA1, BNIPL, CDH2 and GYS increased in SULT2B1-siRNA-treated HPSECs compared with control-siRNA-treated HPSECs. The expression of the tight junction marker claudin-1 was next examined. Claudin-1 immunoreactivity was visible at the membrane, cytoplasm and nucleus in control siRNA-treated cells. Treatment with SULT2B1-siRNA resulted in decreased cytoplasmic and nuclear claudin-1 (Fig. 6b). The protein expression levels of CCND1, CLND1 and CDH1 were also decreased by SULT2B1-siRNA (Fig. 6c). Upon stimulation with 100 ng/mL EGF, the phosphorylated AKT levels decreased in the SULT2B1-siRNA-treated HPSECs compared with the control-siRNA-treated HPSECs (Fig. 6d). As shown in Fig. 6e, 24(R/S),25-EC and 27HC inhibited AKT phosphorylation following 100 ng/mL EGF treatment. The CCND1, CLND1 and CDH1 protein levels were also suppressed by treatment with the two oxysterols (Fig. 6f).

## Discussion

EGF and IGF-1 promote gastric epithelial cell proliferation, epithelization, wound healing, and epithelial development through the PI3K/AKT signaling pathways. The present study suggests that loss of SULT2B1 expression in normal gastric epithelial cells might suppress gastric epithelial development and promote gastric carcinogenesis upon 3-MCA induction. SULT2B1 might enhance PI3K/AKT signaling and thus promote gastric epithelial cell proliferation, epithelization, and epithelial development.

Hyland PL et al. reported that two SNPs in SULT2B1 (rs4149455 and rs1052131) were associated with esophageal squamous cell carcinoma risk [41]. Previous studies have revealed that SULT2B1b was expressed in hepatocellular carcinoma cells and hepatic progenitor cells but not in normal hepatocytes. The SULT2B1b mRNA levels were higher in clinical hepatocellular carcinoma tumor samples than in samples from the nontumorous tissue adjacent to the tumors [39, 42]. The proliferation-promoting ability of SULT2B1b has also been verified in a partial hepatectomy model [43].

In vivo and in vitro data have suggested that SULT2B1 deletion contributed to the gastric carcinogenesis induced by the carcinogenic agent 3-MCA. Under physiological conditions, SULT2B1 was nearly exclusively expressed in the gastrointestinal tract, which contained high levels of oxysterols from either food or cholesterol oxidation. For the first time, the relationship between SULT2B1 expression and gastric epithelial function was studied in the present work. The gastric mucosa is continuously exposed to various exogenous and endogenous irritants. Gastric epithelial cell restitution, proliferation, and development are important components of the mucosal defense system. Growth factors, such as EGF and IGF-1, play important roles in adaptive protection by increasing mucosal cell proliferation and enhancing mucosal lesion healing [4, 44, 45]. The present study revealed that SULT2B1 might promote gastric epithelial cell proliferation and wound healing. Claudin-1 is an integral membrane protein and a component of TJ strands, which are located along the cell membrane at the apical edges and are considered critical for epithelial cell polarity [46]. Current data indicates that SULT2B1 deletion or knockdown decreased claudin-1 expression in intracellular compartments, such as cytoplasm and nucleus. The upregulated and intracellular localization of claudin-1 has been reported to be correlated with cell migration and proliferation [47, 48]. The change in the cellular distribution of claudin-1 in gastric epithelial cells affected by SULT2B1 might be a result of differences in cell proliferation between cells transfected with control or SULT2B1-siRNA.

The PI3K/AKT signaling pathway plays essential roles in normal cellular functions, such as protein synthesis, growth control, and nutrition/energy balance. PI3K/AKT activation by growth factors can stimulate protein synthesis and cell growth [49, 50]. The maintenance of gastric epithelial function relies on the proper functioning of the PI3K/AKT pathway. The present data suggested that SULT2B1 deletion inhibited PI3K/AKT signaling in response to growth factors, but SULT2B1 overexpression promoted this pathway. The attenuated response to growth factors might weaken epithelial cell development during the damage-repair process, thereby priming epithelial cells for a malignant transition upon carcinogenic agent induction. Although SULT2B1 might promote normal epithelial cell proliferation via the PI3K/AKT signaling pathway, the PI3K/AKT signaling pathway is still involved in gastric cancer progression. The PI3K/AKT pathway is a frequently activated pathways in gastric cancer, and PI3K inhibitors have been developed to target this prominent signaling cascade [51]. SULT2B1 plays roles in normal gastric epithelial and gastric tumor cells. Chen W et al. reported that SULT2B1 promoted human gastric cancer cell line proliferation and tumor growth in vitro and in vivo [52]. On the basis of the data from The Cancer Genome Atlas, no statistical difference was observed in SULT2B1 mRNA expression between gastric tumor and normal gastric tissue (Fig. S2A) among tumors from patients with stomach adenocarcinoma in different stages (1–4) and grades (1–3) (Fig. S2B, C). SULT2B1 mRNA expressions were higher in gastric tumors from patients with N3, but not N1 or N2 lymph node metastasis, than normal gastric tissue (Fig. S2D). Kaplan–Meier survival analysis indicated that patients with high expression of SULT2B1 in gastric cancer tissue had a lower survival probability than other patients (Fig. S2E). In addition, by immunohistochemical staining, no difference was observed in SULT2B1 expression between gastric cancer and para-tumor tissues from patients with gastric tumor. The representative images are shown in Fig. S2F of the supplemental data. Therefore, the role of SULT2B1 in the development of gastric cancer is complicated and requires further investigation. The present study emphasized the possible role of SULT2B1 in the malignant transformation of normal gastric epithelial cells, that is, gastric carcinogenesis. SULT2B1 might regulate the function of these cells due to the abundant expression of SULT2B1 in normal gastric epithelial cells, thereby promoting their repair after damage and preventing gastric carcinogenesis induced by the carcinogenic agent 3-MCA.

Oxysterols play important functions in regulating lipid and sterol biosynthesis, generating bile acids as substrates and mediating the return of excess cholesterol to the liver for excretion by reverse cholesterol transport [53]. Despite these beneficial effects, oxysterols are also bioactive molecules that induce adverse effects, such as triggering cellular and molecular insults that lead to foam cell formation in the vascular wall and atherogenesis and promoting cytotoxic, oxidative, and/or inflammatory effects involved in many important degenerative diseases [20]. Thus, oxysterols serve protective and cytotoxic functions depending on the different cell types and various cell microenvironments. Oxysterols serve their functions through binding nuclear receptors (e.g., LXR, ROR, and ERα), GPCRs (e.g., GPR183, CXCR2, and SMO), and regulatory or transport proteins (e.g., oxysterol binding protein) [54]. The underlying mechanisms of oxysterols 24(R/S),25-EC and 27-HC regulating PI3K/AKT signaling and gastric epithelial function should be further investigated.

## Conclusions

The abundant SULT2B1 expression in the normal gastric epithelium might modulate the oxysterol concentration from food or cholesterol oxidation and regulate epithelial development via the PI3K/AKT signaling pathway. This condition might maintain epithelial function and suppress gastric carcinogenesis induced by carcinogenic agents.

## Supplementary information

**Additional file 1: Figure S1.** The TIC of the samples. **Figure S2.** The expression of SULT2B1 in human gastric tumor. **Table S1.** MRM condition, quantitation ranges and linearity data of the samples. **Table S2.** LOD and LOQ data of the samples. **Table S3.** Primer sets used for qRT-PCR.

## Data Availability

The datasets used and analysed during the current study are available from the corresponding author on reasonable request.

## Abbreviations

22(R) HC: Cholest-5-ene-3beta,22R-diol 22(R)-hydroxycholesterol
24(R/S),25-EC: 24(R/S),25-Epoxycholesterol
24(S) HC: 24(S)-Hydroxycholesterol
25HC: Cholest-5-ene-3beta,25-diol (25-hydroxycholesterol)
25HC3S: 25-Hydroxycholesterol-3-sulfate
27HC: 27-Hydroxycholesterol
3-MCA: 3-Methylcholanthrene
4βHC: 4β-hydroxycholesterol
7KETO: 3β-hydroxy-5-cholesten-7-one (7-ketocholesterol)
7αHC: 7α-Hydroxycholesterol
7βHC: 7β-hydroxycholesterol
AKT: Serine/threonine-kinase
Chol: Cholesterol
CS: Cholesterol 3-sulfate
EGF: Epidermal growth factor
IGF-1: Insulin-like growth factor-1
LC-MS: Liquid chromatography and mass spectrometry
PI3K: Phosphatidylinositol-3-kinase
qRT-PCR,: Quantitative real-time polymerase chain reaction
SULT2B1: Hydroxysteroid Sulfotransferase 2B1
SULT2B1^−/−^: SULT2B1-deficient
WT: Wild-type
