# Supplementary material for: Hydroxysteroid sulfotransferase 2B1 affects gastric epithelial function and carcinogenesis induced by a carcinogenic agent
Source: Lipids Health Dis. 2019 Nov 22;18:203. doi: 10.1186/s12944-019-1149-6 (PMC6874824; doi:10.1186/s12944-019-1149-6)
Supplement: Supplementary file 1 — Additional file 1: Figure S1. The TIC of the samples. Figure S2. The expression of SULT2B1 in human gastric tumor. Table S1. MRM condition, quantitation ranges and linearity data of the samples. Table S2. LOD and LOQ data of the samples. Table S3. Primer sets used for qRT-PCR. [file 12944_2019_1149_MOESM1_ESM.docx]

**Supplemental Materia
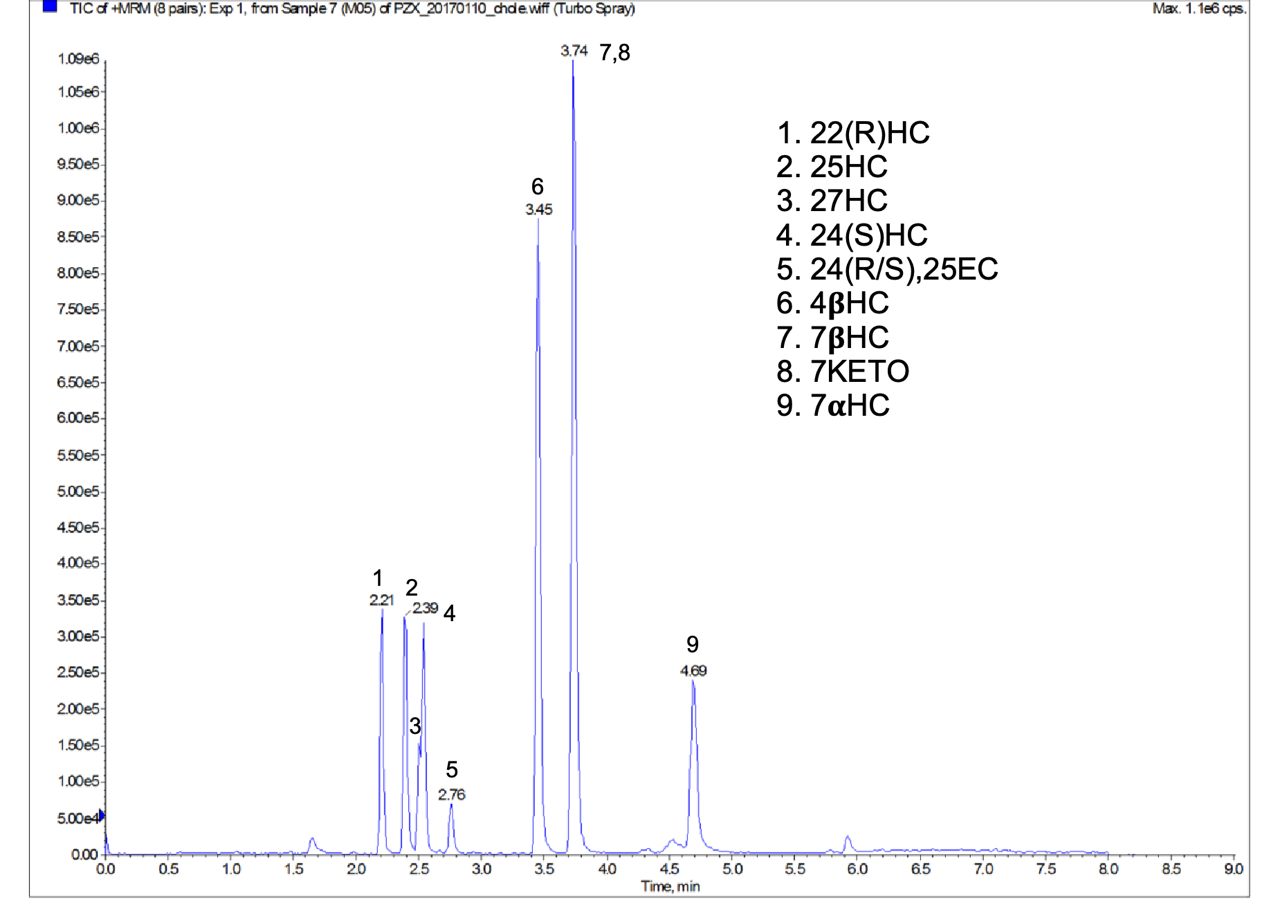
**

**Figure. S1 The TIC of the samples.**


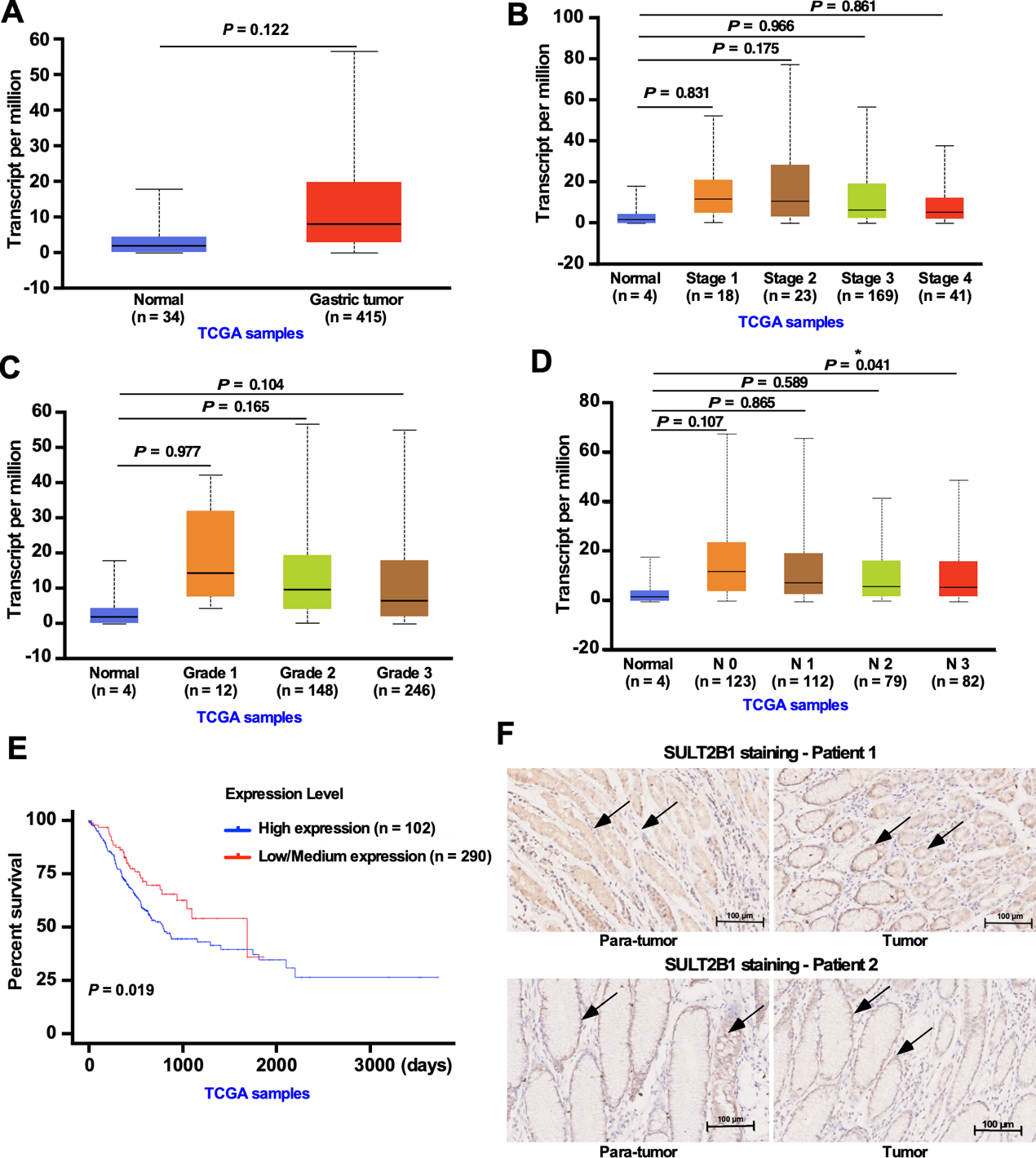
**Figure. S2** **The expression of SULT2B1 in human gastric tumor.**

(A) The expression of SULT2B1 in STAD (Stomach adenocarcinoma) based on sample type. (B) The expression of SULT2B1 in STAD based on individual cancer stage. (C) The expression of SULT2B1 in STAD based on tumor grade. (D) The expression of SULT2B1 in STAD based on nodal metastasis status. (E) Effect of SULT2B1 expression level on STAD patient survival. (F) The immunohistochemical staining of SULT2B1 in gastric tumor tissue and para-tumor tissue from 7 patients with gastric adenocarcinoma, and the representative images were shown. * *P* < 0.05

| Metabolites | Retention  time (min) | Precursor  (*m/z*) | Product  (*m/z*) | DP  (V) | CE  (V) | Linearity range of mixed  standards (ng/mL) ^a^ | Coefficient of determination ^b^ |
| --- | --- | --- | --- | --- | --- | --- | --- |
| 22(R)HC | 2.20 | 385.3 | 367.3 | 130 | 20 | 0.85-617 | 0.9999 |
| 25HC | 2.40 | 385.3 | 161.2 | 130 | 20 | 0.85-617 | 1 |
| 27HC | 2.52 | 385.3 | 161.2 | 130 | 27 | 0.85-617 | 0.9998 |
| 24(S)HC | 2.57 | 367.3 | 159.2 | 130 | 32 | 0.85-617 | 0.9999 |
| 24(R/S),25-EC | 2.76 | 385.3 | 161.2 | 130 | 30 | 0.85-617 | 0.9996 |
| 4βHC | 3.45 | 385.3 | 367.3 | 130 | 20 | 0.85-206 | 0.9999 |
| 7βHC | 3.74 | 385.3 | 367.3 | 130 | 20 | 0.85-69 | 0.9996 |
| 7KETO | 3.85 | 401.3 | 175.2 | 130 | 35 | 0.85-69 | 0.9996 |
| 7αHC | 4.69 | 385.3 | 367.3 | 130 | 20 | 0.85-69 | 0.9995 |

**Table. S1 MRM condition, quantitation ranges and linearity data of the samples.**

^a^ Stock solution of each oxysterol standard was freshly prepared in methanol (1 mg/mL). The mixed standard solution (50 μg/mL) was obtained by adding 50 μL of each solution described above. 8 calibration concentrations with 0.85, 2.54, 7.62, 22.86, 68.59, 205.76, and 617.28 μg/mL were prepared by serial dilution of stock solution for linear range determination.

^b^ Coefficient of determination (R^2^) were calculated for linearity range at the concentration listed here. R^2^ close to 1 indicates the perfect correlation between x and y, which are two variables in the linear regression equation of y = ax + b.

| Metabolites | LOD (ng/mL) ^a^ | LOQ (ng/mL) ^b^ |
| --- | --- | --- |
| 22(R)HC | 0.84 | 2.80 |
| 25HC | 0.66 | 2.20 |
| 27HC | 0.75 | 2.50 |
| 24(S)HC | 2.28 | 7.60 |
| 24(R/S),25-EC | 1.08 | 3.60 |
| 4βHC | 1.26 | 4.20 |
| 7βHC | 0.63 | 2.10 |
| 7KETO | 0.26 | 0.85 |
| 7αHC | 0.54 | 1.80 |

**Table. S2 LOD and LOQ data of the samples.**

^a^ LOD is estimated according to the equation of x_LOD_ = 3.9 × s_y,b_ ÷ b (x_LOD_: Limit of detection; s_y,b_: Standard deviation of the blank (pseudo-blank) signals; b: Slope of calibration curve).

^b^ LOQ is estimated according to the equation of x_LOQ_ = 3.3 × x_LOD_.

**Table. S3 Primer sets used for qRT-PCR**

| Gene name | Primer | Sequence (5’-3’) |
| --- | --- | --- |
| human SULT2B1 | Forward | CAGTTTGGCTCCTGGTTCG |
|  | Reverse | GAGGCAGCAGCGTGTAGTTG |
| human VIM | Forward | GAACGCAAAGTGGAATC |
|  | Reverse | GTCAGGCTTGGAAACAT |
| human CDH1 | Forward | GCGGCTGATACTGACCC |
|  | Reverse | GATTGCCCCATTCGTTC |
| human ZO-1 | Forward | GATGAACGGGCTACGC |
|  | Reverse | ACCGCTGGTCAGGAGAT |
| human PGA3 | Forward | AAGTGGCTCGTCGTTCT |
|  | Reverse | TTGGGCTGTATGGGAAC |
| human CA5a | Forward | TTGGAAAACCACTACAGA |
|  | Reverse | ACACGCCTATCACAGC |
| human FHIT1 | Forward | CAGAACTGTCCTTCGCTC |
|  | Reverse | TTCCACCACTGTCCCG |
| human CCND1 | Forward | GCGAGGAACAGAAGTGCG |
|  | Reverse | GGATGGAGTTGTCGGTGTAGA |
| human C-Met | Forward | TACCACTCCTTCCCTG |
|  | Reverse | ATTTTCATTGCCCATT |
| human RUNX3 | Forward | GCAGGCAATGACGA |
|  | Reverse | GTCAGGGTGAAACTCTT |
| human KRAS | Forward | TGTCCGTCAGAACCCATGC |
|  | Reverse | AAAGTCGAAGTTCCATCGCTC |
| human BRCA1 | Forward | AATACAAGAGCGTCCC |
|  | Reverse | CCGTTTGGTTAGTTCC |
| human BNIPL | Forward | GACCTGGAGATAGACGAAT |
|  | Reverse | AGGGTACGGTAACACTGAC |
| human CK18 | Forward | TGACCGTGGAGGTAGAT |
|  | Reverse | CTCTGTGAGCGTCGTCT |
| human BCL2L11 | Forward | AGAGTTGCGGCGTATTGGA |
|  | Reverse | ATTCGTGGGTGGTCTTCG |
| human CDX2 | Forward | CGCAGAACTTCGTCAG |
|  | Reverse | GCGTAGCCATTCCAGT |
| human P53 | Forward | TCCTCAGCATCTTATCC |
|  | Reverse | CAGTCAGAGCCAACCT |
| human HK1 | Forward | TGGCTCCGAAATGTGATG |
|  | Reverse | CAGTGCGAATGTCGTTGA |
| human GYS | Forward | ACCGCACTTTGTCCAT |
|  | Reverse | ACCCACCTTGTTAGCC |
| human TDGF1 | Forward | AACTGTGAGCACGATG |
|  | Reverse | GGTAGAAATGCCTGAG |
| human OCLN | Forward | TGGAAGCGGTTTAGGAA |
|  | Reverse | GCGATGAAGATGATGGC |
| human CCNA2 | Forward | TTGGATAATCAAGAGGGACC |
|  | Reverse | TGTCACCGTTCCTCCTTG |
| mouse SULT2B1 | Forward | TGTCACCGTTCCTCCTTG |
|  | Reverse | TTGGATAATCAAGAGGGACC |
